# Health Disparities and Differences in Health-Care-Utilization in Patients With Pulmonary Arterial Hypertension

**DOI:** 10.3389/fpsyt.2022.813506

**Published:** 2022-02-22

**Authors:** Da-Hee Park, Tanja Meltendorf, Kai G. Kahl, Jan C. Kamp, Manuel J. Richter, Marius M. Hoeper, Karen M. Olsson, Jan Fuge

**Affiliations:** ^1^Department of Respiratory Medicine, Hannover Medical School, Hannover, Germany; ^2^Biomedical Research in Endstage and Obstructive Lung Disease Hannover (BREATH), German Center for Lung Research (DZL), Hannover, Germany; ^3^Department of Psychiatry, Social Psychiatry and Psychotherapy, Hannover Medical School, Hannover, Germany; ^4^Department of Internal Medicine, Universities of Giessen and Marburg Lung Center (UGMLC), Justus Liebig University Giessen, Giessen, Germany; ^5^German Center for Lung Research (DZL), Giessen, Germany

**Keywords:** pulmonary arterial hypertension, mental health, quality of life, health disparities, health-care-utilization

## Abstract

**Introduction:**

Mental disorders are common in patients with pulmonary arterial hypertension (PAH) and contribute to impaired quality of life (QoL). The impact of mental disorders on access to health care, differences in clinical parameters and treatment in patients with PAH is unclear. In this study we sought to assess the impact of mental disorders and other health disparities on health-care-utilization in patients with PAH.

**Methods:**

In a cross-sectional observational study of patients with PAH, mental disorders were characterized using a structed clinical interview. In addition, patients completed a self-administered questionnaire to assess QoL, symptoms of anxiety and depression, lifestyle-factors and educational status. Number of outpatient visits and communication events per year were calculated as a surrogate for health-care-utilization and were compared by the presence of mental disorder. Linear regression analysis was conducted to assess the impact on health-care-utilization.

**Results:**

117 patients with PAH participated in this study (70% female, median age 59 (interquartile range, 49–70) years). Significant differences between patients with or without mental disorders were found in anxiety, depression and QoL. There were no significant differences in clinical parameters. Patients with mental disorders had higher rates of outpatient visits and communication events than patients without mental disorders. Linear regression revealed a gain of 2.2 communication events per year in the presence of any mental disorders.

**Conclusion:**

Mental disorders in patients with PAH are common and significantly affect health-care-utilization. This higher demand in patients with mental disorder needs to be addressed by physicians, psychiatrists and specialized nurses offering therapeutic strategies.

## Introduction

Pulmonary arterial hypertension (PAH) is a rare disease characterized by increased pulmonary vascular resistance due to remodeling of pulmonary vasculature (1, 2). Symptoms of PAH include clinical signs of heart failure, dyspnea on exertion, syncope leading to impaired physical activities and a reduced quality of life (QoL) (3). Therapy of PAH has greatly advanced over the last decades improving clinical outcomes and survival of PAH patients. However, PAH remains a fatal illness with right heart failure being the primary cause of death (4).

Mental disorders such as major depression disorder (MDD) or panic disorder (PD) are common in patients with PAH, and patient's quality of life, mental health, and access to medical care have been addressed in recent studies (5–10). The prevalence of anxiety, depression and adjustment disorders in patients with PAH correlate with disease progression, intensity of symptoms as well as functional impairment (11, 12). Mental disorders such as major depression disorder, panic disorder or adjustment disorder are common in patients with PAH (13).

Health disparities are defined as significant differences in health or outcomes with correlation to greater obstacles to healthcare based on ethnicity, sex, age, socioeconomic status, occupation, mental health, cognitive or physical disability (14). Health disparities can impact quality of life, disease treatment and outcomes (15, 16). With only few studies addressing health disparities in patients with PAH, the American Thoracic Society encouraged further research to achieve more equitable care, improve advocacy efforts and public health policy for patients with PAH (8). Health disparities, in particular mental disorders may contribute to differences in clinical parameters, treatment and survival in patients with PAH. Thus, in this study, we sought to assess possible implications of mental disorders in patients with PAH leading to differences in PAH treatment and health-care utilization.

## Methods

This cross-sectional observational study included patients with a confirmed diagnosis of PAH from one PH referral center (Hannover Medical School in Germany). This patient cohort is a subsample of a larger cohort that was originally described by Olsson et al. (13). All patients gave written informed consent and the study was approved by the local institutional review board (Nr. 8540_BO_K_2019). Patients were contacted and interviewed between September 2019 and March 2020 to perform a structured clinical interview. This study further comprised self-administrated questionnaires and data was enriched using a clinical research database.

### Patient Setting and Clinical Parameters

Patients were selected from a clinical research database based on diagnosis of PAH according to current criteria (1) and age ≥ 18 years. Assessment included hemodynamics from right heart catherization at time of diagnosis, 6-minute walk distance (6MWD), serum levels of N-terminal fragment of pro-brain natriuretic peptide (NT-proBNP), WHO functional class (FC) and diffusing capacity of the lung for carbon monoxide (D_LCO_). Lifestyle factors included smoking status (active smoker, former smoker or never-smoker), duration of smoking and mean number of cigarettes per day to calculate the number of packyears (17) as well as alcohol abuse as drinks per week.

### Assessment of Mental Disorders, Quality of Life and Educational Status

Psychiatric characterization was performed using the Structured Clinical Interview (SCID) for Diagnostic and Statistical Manual of Mental Disorders, fifth Edition (DSM-V) by a medical student carefully trained and under the supervision of a senior physician to determine the presence of MDD and PD and covered the past 4 weeks prior to the interview (18). The Hospital Anxiety and Depression Scale (HADS) was used to assess symptoms of anxiety and depression (19). The HADS questionnaire comprises two subscales for anxiety (HADS-A) and depression (HADS-D), each containing 7 questions for a maximum of 21 points per subscale. Higher scores indicating a more severe anxiety or depression. A score > 11 per subscale was proposed by Bjelland et al. and Lowe to be associated with significant anxiety or depression while a cut-off score of > 8 for both subscores showing probable signs of anxiety or depression (20, 21). The World Health Organization Quality of Life [WHO-QoL-BREF (22)] questionnaire was used to assess quality of life. To assess patients educational status, the sociodemographic standards of the German Federal Statistical Office (destatis) (23) were applied. Further educational status was categorized in low (no education), moderate (non-academic education) and high (academic education) educational status.

### Assessment of Outpatient Visits and Patient Communication

Total number of outpatient visits and patient communication datasets were derived from a clinical research database as well as the date from first and last outpatient visit or communication event respectively. Patient communication is defined as any kind of unscheduled communication event (e.g., e-mail, mail, phone calls or other correspondence) documented in the clinical research database by a physician or a specialized nurse. Time-variables comprising the time in years between first and last date of outpatient visit or communication event were calculated and set into relation to the number of visits and communications. This allowed us to calculate outpatient visits and communication events per year for each patient.

### Statistical Analysis

IBM SPSS Statistics (version 28.0, IBM Corp., Armonk, New York) and Stata 13.0 (State Corp LP, College Station, Texas, USA) statistical software programs were used for statistical analysis. Continuous parameters are presented as median and interquartile range (Q_25_-Q_75_) or as mean and standard deviation (SD). Categorical variables are presented as number (n) and percent (%). Comparisons of continuous parameters were conducted using *t*-test or Mann-Whitney *U* test and comparison of categorical parameters by using Chi-square-test or fisher's exact test, unless indicated otherwise. Linear regression models calculated on dependent variables outpatient visits per year and correspondences per year in a stepwise forward setting. Independent variables were age, sex, packyears, HADS-A and HADS-D scores, QoL, education, WHO FC, 6MWD and the diagnosis of any MDD or PD. All tests were two-sided, *p*-values < 0.05 were considered statistically significant.

## Results

A total of 117 patients were enrolled in this study. Patient characteristics are shown in Table 1. The majority of patients were female (n=82, 70%); median age was 59 (interquartile range, 49–70) years. Twenty six patients presented with MDD and 10 patients with PD resulting in 31 (27%) patients (five patients had a combination of MDD or PD). All patients were treated with PAH medications, most of them (73%) with combination therapy. Disease severity indicated by WHO FC, 6MWD, NT-proBNP, DLCO, hemodynamics at time of diagnosis as well as PAH treatment did not differ between patients with or without mental disorders (Table 1). In addition, there were no differences in drinking habits or exercise scores but patients with any form of mental disorder were nearly two-times more likely to be active smokers (23 vs. 12%). Patients with MDD or PD had significantly worse HADS-A and HADS-D scores. Psychological and physical QoL was worse in patients with MDD or PD (all *p* < 0.001).

**Table 1:** Characteristics of the patients at baseline.

	**All patients** ***n* = 117**	**Patients with** **depression or panic** **disorder** ***n* = 31 (27%)**	**Patients without** **depression or panic** **disorder** ***n* = 86 (74%)**	***p*-value**
Age (years)	59 (49–70)	51 (40–60)	61 (50–70)	**0.010**
Female sex (%)	82 (70%)	22 (71%)	60 (70%)	0.900
BMI (kg/m^2^)	25 (22–31)	25 (23–33)	25 (22–30)	0.369
Diagnosis				
→ IPAH, n (%)	58 (50%)	13 (42%)	45 (52%)	0.248^a^
→ HPAH, n (%)	8 (7%)	4 (13%)	4 (5%)	
→ Associated PAH, n (%)	51 (44%)	14 (45%)	37 (43%)	
Time since PAH diagnosis (years)	5 (2–10)	6 (2–9)	5 (3–10)	0.395
WHO FC				
→ I/II, n (%)	58 (50%)	12 (38%)	46 (53%)	0.469^a^
→ III, n (%)	53 (45%)	17 (55%)	36 (42%)	
→ IV, n (%)	6 (5%)	2 (7%)	4 (5%)	
6MWD (m)	429 (340–503)	429 (335-500)	431 (345–505)	0.700
NT-proBNP (ng/l), *n* = 105	236 (101–691)	248 (67–1128)	228 (105–505)	0.222
DLCO (% pred.), *n* = 75	62 (45–74)	65 (50–73)	58 (39–74)	0.263
paO_2_, mmHg	67 (60–77)	68 (61–76)	66 (57–78)	0.342
Hemodynamics at diagnosis				
→ mPAP (mmHg)	45 (37–55)	45 (41–55)	46 (37–55)	0.945
→ PAWP (mmHg)	9 (6–12)	8 (6–11)	9 (6–12)	0.175
→ CI (l/min/m^2^)	2.4 (2.1–2.9)	2.5 (2.2–3.0)	2.4 (2.0–2.9)	0.552
→ PVR (dyn·s·cm^−5^)	652 (464–830)	642 (481–891)	652 (448–824)	0.819
PAH medication				
→ Monotherapy	32 (27%)	7 (23%)	25 (29%)	0.082^a^
→ Double combination therapy	63 (54%)	14 (45%)	49 (57%)	
→ Triple combination therapy	22 (19%)	10 (32%)	12 (14%)	
Smoking Status				
→ Active, n (%)	17 (15%)	7 (23%)	10 (12%)	0.267^a^
→ Former, n (%)	9 (8%)	3 (10%)	6 (7%)	
→ Never, n (%)	91 (78%)	21 (68%)	70 (81%)	
→ Packyears	15 (10–25)	12 (9–20)	20 (10–40)	0.161
Sociodemographic Items				
→ Drinking (drinks per week)^b^	0.74 ± 1.31	0.71 ± 1.22	0.74 ± 1.35	0.901
→ Exercise Score (points)	3 (2–4)	2 (2–3)	3 (2–4)	0.126^c^
→ HADS-A (points)	6 (3–9)	9 (7–12)	5 (2–7)	**<** **0.001**
→ HADS-D (points)	5 (2–7)	8 (5–13)	4 (2–6)	**<** **0.001**
→ QoL-overall (points)	50 (38–75)	38 (25–63)	63 (50–75)	**<** **0.001**
→ QoL-psych (points)	67 (58–79)	54 (46–67)	75 (63–88)	**<** **0.001**
→ QoL-physical (points)	57 (41–75)	43 (32–64)	66 (50–75)	**<** **0.001**
Education				
→ Low	19 (16%)	3 (10%)	16 (19%)	0.347^a^
→ Moderate	82 (70%)	22 (71%)	60 (70%)	
→ High	14 (14%)	6 (19%)	10 (12%)	

*Significant p-values are presented in bold*.

*Continuous variables are stated as median and interquartile ranges (IQR) and categorical variables are stated as n and percent (%), unless indicated otherwise*.

a *Chi^2^-test*.

b *Mean and standard deviation (SD) because of distribution of the data*.

c *Non-parametric Mann-Whitney-U-Test because of ordinal scale of the variable*.

*BMI, body mass index; QoL, quality of life; HADS-A, hospital anxiety and depression scale – anxiety; HADS-D, hospital anxiety and depression scale – depression; I/HPAH, idiopathic or heritable pulmonary arterial hypertension; WHO FC, World Health Organization Functional Class; 6MWD, six-minute walking distance; BNP, brain natriuretic peptide; NT-proBNP, N-terminal fragment of pro-brain natriuretic peptide; DLCO, diffusion capacity of the lung for carbon monoxide; paO_2_, mmHg, arterial pO_2_; mPAP, mean pulmonary arterial pressure; PAWP, pulmonary artery wedge pressure; CI, cardiac index; PVR, pulmonary vascular resistance; SD, standard deviation; IQR, inter quartile range*.

### Outpatient Visits and Communication of Patients With or Without MDD or PD

**Figure 1:**
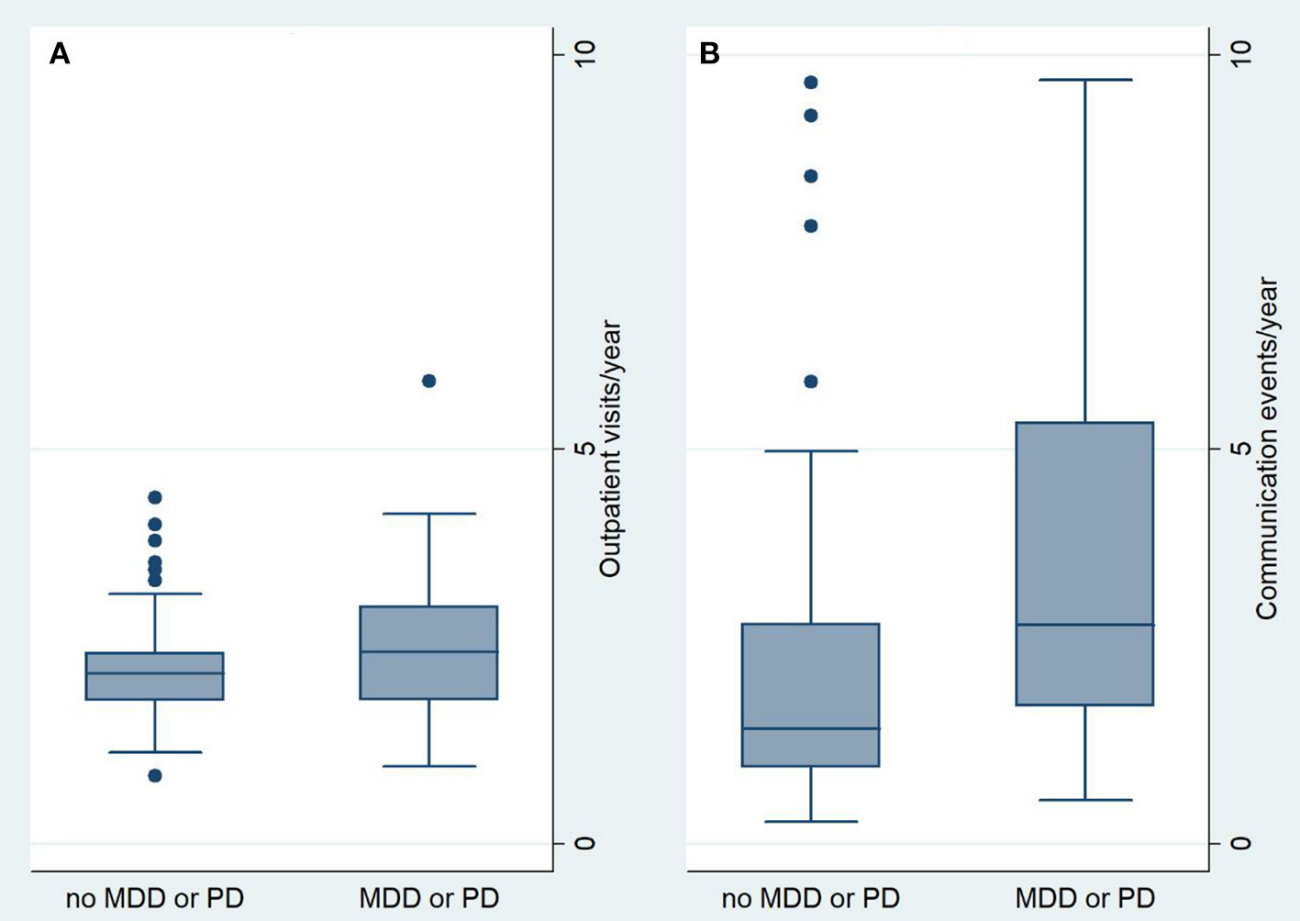
Comparison of Outpatient visits/year **(A)** and communication events/year **(B)**. MDD, major depressive disorder; PD, panic disorder.

Detailed results are stated in Table 2. Grouped together, the patients had a median number of 2.2 outpatient visits and 1.8 communication events per year. Comparison of visits and communication revealed significant differences between patients with and without MDD or PD. Patients with MDD or PD had significantly more outpatient visits per year compared to patients without MDD or PD (2.4 vs. 2.2 visits per year, *p* = 0.022, Figure 1A). The number of communication events also differed significantly (Figure 1B), with 2.8 (interquartile range, 1.8–6.2) communication events per year in patients with MDD or PD compared to 2.1 (interquartile range, 1.0–2.9) communication events in patients without MDD or PD (*p* = 0.001).

**Table 2:** Comparison of patient contacts and communication events by mental disorders.

**Item**	**All PAH** ***n* = 117**	**Patients with** **depression or** **panic disorder** ***n* = 31 (27%)**	**Patients** **without depression or** **panic disorder** ***n* = 86 (74%)**	***p-*value**
Patient contacts				
→ Outpatient visits	2.2 (1.8–2.6)	2.4 (1.8–3.0)	2.2 (1.8–2.4)	**0.022**
→ Communication events	1.8 (1.0–3.6)	2.8 (1.8–6.2)	1.5 (1.0–2.9)	**0.001**

*Significant p-values are presented in bold*.

*Continuous variables are stated as median and interquartile ranges (IQR) and categorical variables are stated as n and percent (%), All p-values were derived using t-test*.

*PAH, pulmonary arterial hypertension; IQR, inter quartile range*.

### Impact on Number of Outpatient Visits and Communication Events

Impact on number of outpatient visits and communication events was tested using clinical parameters, the presence of MDD or PD, QoL and lifestyle factors. Linear regression analysis showed a significant impact of age and 6MWD on the number of visits per year. Per 10 years of patients age there was a loss of 0.2 visits per year (*p* = 0.003) and per 50 m 6MWD there was a loss of 0.1 visits per year (*p* = 0.007). Impact on communication events per year was only determined by the presence of MDD or PD. If a patient had any current MDD or PD, correspondence increased by 2.2 communication events per year (*p* = 0.001) with the linear regression analysis stating a constant of 2.4 communication events per year. This results in a near two-fold number of communication events in the presence of MDD or PD. The other covariates sex, packyears, HADS-A and HADS-D scores, QoL, education and WHO FC had no significant impact on the number of outpatient visits or communication events per year.

## Discussion

The main finding of this study can be summarized as follows: patients with MDD or PD have a higher need for patient care with a higher demand for extracurricular communication. The presence of MDD or PD was the only factor significantly influencing the rate of communication events. Patients with MDD or PD had worse QoL and HADS-scores while no differences in PAH medication or lifestyle factors were found. Education had no impact on number of outpatient visits or communication events nor were there any differences in education between patients with or without MDD or PD.

Impact on outpatient visits was determined by age and 6MWD while communication events were impacted by the presence of MDD or PD. While the association of age and 6MWD on outpatient visits was significant, absolute differences in number of outpatient visits were small suggesting only limited impact on clinical practice. Overall, the difference of 0.2 or 0.1 visits per year respectively might not result in an impaired health-care-utilization for patients with MDD or PD.

The only determinant for an increased number of communication events was the presence of MDD or PD. These patients had an almost two-fold increase in communication events while there were no differences in therapy strategies or disease severity (Table 1). This is in line with previous studies showing that the presence of a mental health disorder is associated with significantly higher health care resource utilization and costs independent of disease severity (24, 25).

Communication events beyond outpatient visits seem to be a valuable instrument for patients with mental disorders to facilitate health-care-utilization. Recent data have shown that the use of telecommunications such as video consultations reduced the need for onsite outpatients visits with increased use during the COVID-19 pandemic (26). While established patients had no worsening of mental disorders during the Covid-19 pandemic (27), health care utilization in form of diagnostic delay and increased time to referral in patients with PAH was impaired (28). Thus, future strategies and technologies are needed to fulfill patient's health-care-needs.

Our findings raise questions about optimizing the management of patients with PAH especially in the setting of mental disorders. First, mental disorders such as major depressive disorders or panic disorders need to be assessed in routine care. However, a SCID maybe not be feasible for screening purposes. The HADS was recently proven to detect anxiety and depression in patients with PAH (13) but also other tools such as the Hamilton Anxiety- or Depression Scale (HAM-A or HAM-D) might be feasible. Further, studies have shown that poor access to coordinated mental health care had impact on acute health-care-utilization. Different models of care such as a multidisciplinary approach with the integration of medical and psychiatric health care have been described in primary as well as specialty care such as is PAH care (29). Recent studies have shown a higher prevalence of adjustment disorders among mental disorders in patients with PAH (13). Metacognitive treatment, a cognitive behavioral therapy, has been successfully applied to a patient with newly diagnosed PAH suffering from severe adjustment disorder (30, 31). With only limited data on psychiatric treatment options in patients with PAH, further studies are needed. This preliminary report on health disparities and health care utilization raises the question of improved patient care for patients with PAH. Knowledge, deeper understanding, and education of psychiatric disorders is key to prevent further stigma, increase awareness for patients challenges and needs and improve QoL beyond the physical burden of PAH. We suggest a specialized approach to address the needs of patients with MDD or PD through available treatment programs. PAH patients with MDD or PD have an increased need for assistance of social workers, physiotherapist and occupational therapist which should be a valuable asset in PAH patient's care. Further the availability of fast-track psychiatric consultations and acute crisis management should be of importance. In summery we suggest a comprehensive multidisciplinary psychiatric medical care for patients with MDD or PD and conclude that further research is needed to inform clinicians on the differences in health care utilization in the presence of psychiatric disorders.

### Limitations

This study has limitations and strength. One limitation is the monocentric cross-sectional design of the study and the use of outpatients visits and another that communication events per year as a surrogate for health-care-utilization has not been validated. Further no economical evaluation has been conducted. However, for a rare disease the analyzed sample size is high and the SCID-derived diagnosis of mental disorders is considered the gold standard of psychiatric assessment.

## Conclusion

Compared to PAH patients without mental disorders, patients with MDD or PD are more likely to seek communication with their health care provider and health care systems should address the higher need of patients with MDD or PD. These findings were independent of disease severity or therapy. Future studies are needed to inform clinicians, psychiatrists and specialized nurses on differences in health care utilization as well as patients' individual needs.

## Data Availability Statement

The raw data supporting the conclusions of this article will be made available by the authors, without undue reservation.

## Ethics Statement

The studies involving human participants were reviewed and approved by Hannover Medical School. The patients/participants provided their written informed consent to participate in this study.

## Author Contributions

DHP was responsible for study design, implementation of the study, data collection, data interpretation and drafting the manuscript. TM was responsible for data collection and revising the manuscript. KK was responsible for study design, data interpretation, and revising the manuscript. JK was responsible for data interpretation and revising the manuscript. MR was responsible for implementation of the study and critically revising the manuscript. MH was responsible for implementation of the study, data interpretation, and critically revising the manuscript. KO was responsible for study design, implementation of the study, data interpretation, and critically revising the manuscript. JF was responsible for study design, implementation of the study, data collection, statistical analysis, data interpretation, and drafting the manuscript. All authors contributed to the article and approved the submitted version.

## Conflict of Interest

DHP has received honoraria for lectures and/or consultations from Janssen. KK has received honoraria for consultations and/or lectures from Eli Lilly, Janssen, Lundbeck, Neuraxpharm, Otsuka, Pfizer, Servier, Schwabe, Takeda, and Trommsdorff/Ferrer, Alexion, and CannaXan (advisory board). MH has received honoraria for lectures and/or consultations from Acceleron, Actelion, Bayer, GSK, Janssen, MSD and Pfizer, all outside the present study. KO has received honoraria for lectures and/or consultations from Acceleron, Actelion, Bayer, GSK, Janssen, MSD, United Therapeutics and Pfizer, all outside the present study. The remaining authors declare that the research was conducted in the absence of any commercial or financial relationships that could be construed as a potential conflict of interest.

## Publisher's Note

All claims expressed in this article are solely those of the authors and do not necessarily represent those of their affiliated organizations, or those of the publisher, the editors and the reviewers. Any product that may be evaluated in this article, or claim that may be made by its manufacturer, is not guaranteed or endorsed by the publisher.

## Abbreviations

6MWD: 6-minute walk distance
DLCO: diffusing capacity of the lung for carbon monoxide
DSM-V: Diagnostic and Statistical Manual of Mental Disorders V
FC: World Health Organization Functional Class
HADS: Hospital Anxiety and Depression Scale
NT-proBNP: N-terminal fragment of probrain natriuretic peptide
PAH: pulmonary arterial hypertension
QoL: quality of life
SCID, structed clinical interview for DSM-V: Structured Clinical Interview
SD: standard deviation.
